# Severe tricuspid regurgitation due to valvular entrapment of an inferior vena cava stent

**DOI:** 10.1002/ccr3.812

**Published:** 2017-01-17

**Authors:** Kirstin Hesterberg, Ashok Babu, Maria Frank, Shea Hogan, Mori J. Krantz

**Affiliations:** ^1^ Exempla Saint Joseph HospitalDenverColoradoUSA; ^2^ University of Colorado, Cardiothoracic SurgeryAurora, ColoradoUSA; ^3^ Denver Health Medical CenterDenverColoradoUSA

**Keywords:** Inferior vena cava, severe tricuspid regurgitation, stent migration, transthoracic echocardiography, tricuspid valve entrapment

## Abstract

Endovascular venous stenting is increasingly performed for a variety of conditions. Inferior vena cava stent migration has been reported up to 6 months after placement; stent migration 6 months after implantation is uncommon. To our knowledge, this is only the second reported case of late stent migration with valve entrapment 1.

## Introduction

A 73‐year‐old male presented with sepsis with suspected abdominal source. He was found to have migration of inferior vena caval stent, with valve entrapment, a rare complication. Surgical removal was indicated given valve involvement, but other locations of migration may be amenable to percutaneous removal.

## Case Presentation

A 73‐year‐old male physician presented to our hospital with sudden onset fever, fatigue, and upper quadrant abdominal pain, which started on the day of admission. His past medical history was significant for stage III clear cell variant neuroendocrine tumor of the pancreas, with Whipple procedure performed. He developed subsequent ascites and lower extremity edema, which was attributed to his prior procedure. IVC stent was placed approximately 6 months before presentation, with the goal of relieving IVC stenosis, secondary to prior surgical intervention. He had no history of cardiovascular or valvular disease.

Vital signs revealed a temperature of 38.6°C, pulse rate of 51 beats/min, blood pressure 93/59 mmHg, and a respiratory rate 15 breaths/min. Examination was notable for a grade 3/6 systolic murmur over the lower left sternal border, which increased with inspiration. Mild jugular venous pressure elevation was noted with prominent V‐waves. Lungs were clear bilaterally. His abdomen was soft and nontender with no evidence of organomegaly or a pulsatile liver. Moderate lower extremity pitting edema was noted to the upper thighs bilaterally. Laboratory results included a white blood cell count of 12.2 k/mL, hemoglobin of 13.3 g/dL, platelets of 63 k/mL, creatinine of 1.12 mg/dL, glucose of 135 mg/dL, calcium of 7.5 mg/dL, lactate of 1.7 mmol/L, and an international normalized ratio that was mildly elevated at 1.42.

**Figure 1:**
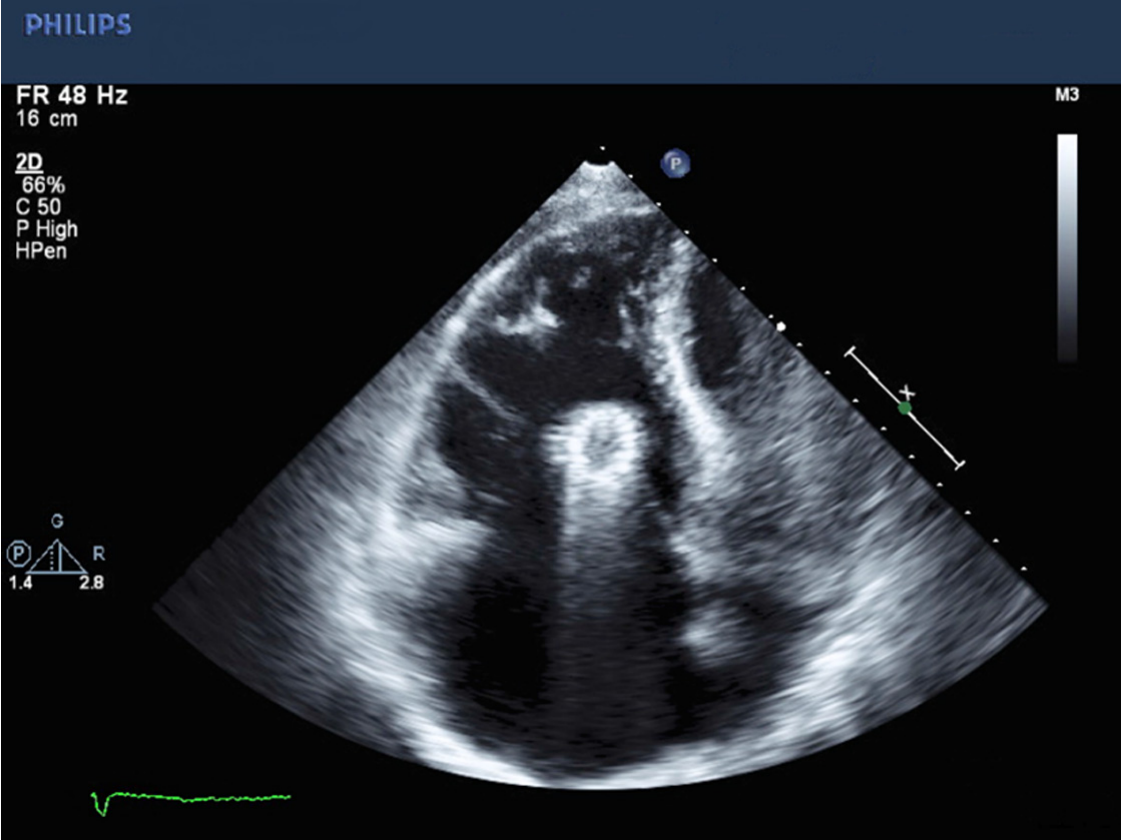
Modified apical four‐chamber view showing entrapment of the stent within the tricuspid valve.

**Figure 2:**
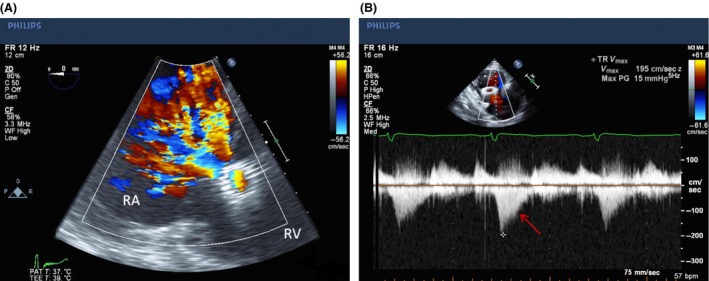
Transesophageal echocardiogram showing severe tricuspid regurgitation by color Doppler (A) and cutoff sign by continuous Doppler (Arrow, B).

**Figure 3:**
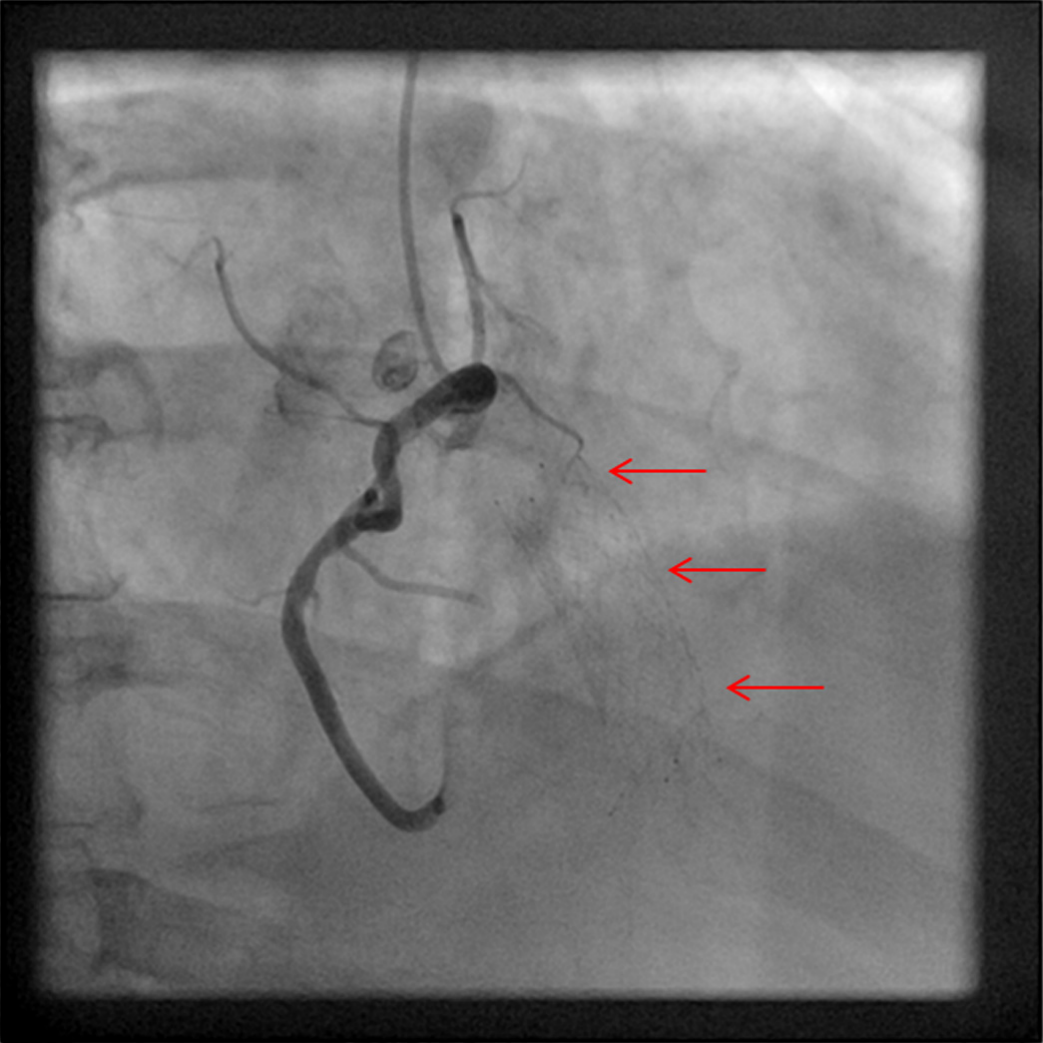
Right anterior oblique coronary angiography image demonstrating no significant atherosclerotic disease and juxtaposition of stent across the tricuspid valve (arrows).

**Figure 4:**
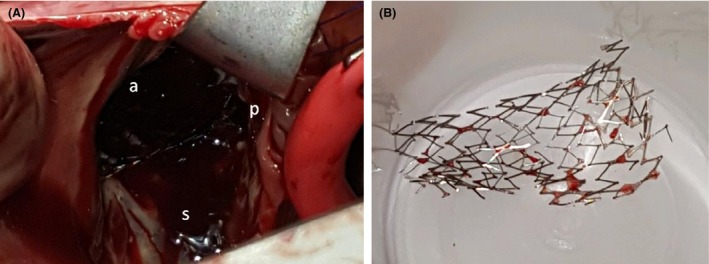
Intraoperative confirmation of stent crossing tricuspid valve (A), with disruption of anterior (a), posterior (p), and septal (s) leaflets. Postsurgical removal of the stent with evidence of missing (retained) struts that were nonextractable (B).

Given the presence of a new systolic murmur and concern for infective endocarditis, a transthoracic echocardiography (TTE) examination was performed (Fig. 1). This revealed the presence of a cylindrical, echo‐dense structure embedded longitudinally within the tricuspid subvalvular apparatus, suggestive of IVC stent migration and entrapment. Left ventricular size and systolic function were normal. The right ventricle was mildly dilated with severe right atrial dilation. Color Doppler demonstrated severe tricuspid regurgitation (Fig. 2A) as well as pulse‐wave Doppler evidence of a “cutoff sign” indicative of rapid right atrial pressure equalization (Fig. 2B). Given entrapment in the valvular apparatus, the patient was not considered an optimal candidate for percutaneous device retrieval. Preoperative coronary angiography was negative for evidence of obstructive coronary artery disease (Fig. 3), but did demonstrate IVC stent migration into the atrioventricular valve plane adjacent to the visualized right coronary artery. The patient underwent surgical stent removal 3 days after admission. Stent position across the tricuspid valve was confirmed intraoperatively (Fig. 4A). The stent was removed largely intact (Fig. 4B), although some of the stent struts remained entrapped within the native valve. His postoperative course was uncomplicated, and repeat echocardiogram revealed only mild tricuspid regurgitation. One month after surgery, he presented with severe lower extremity edema. Computed tomography imaging revealed inferior vena cava stenosis, and a second IVC stent was placed with subsequent resolution of lower extremity edema.

## Discussion

Endovascular venous stenting, particularly IVC filter placement, has increased dramatically over the last 25 years, with an estimated 49,000 placed in 1999 compared to 2000 placed in 1979 2. These stents are placed to treat a wide variety of conditions including reduction in the risk of pulmonary arterial embolization, to improve portal hypertension, to improve venous return to the heart from May–Thurner syndrome or iliac vein/IVC stenosis, to relieve brachiocephalic vein obstruction, and in the setting of dialysis graft outflow stenosis. Extensive literature exists regarding IVC filter migration, with various locations described after migration. Most prior cases of IVC stent migration describe significant hemodynamic compromise (hypotension) or arrhythmias with migration, whereas in the current case, fatigue, fever, and a new murmur were the presenting symptoms, with clinical concern for infective endocarditis. Previously published cases of IVC stent migration report this complication occurring within 24 h after placement 3, 4 and 3 months after placement 5. In the cases of acute migration, one patient reported chest pain, while the other patients exhibited significant hemodynamic compromise, including asystole.

Available reported data suggest a 2–3% migration rate for IVC filters 6, although this percentage is likely an underestimation. In a review of 98 published cases of filter migration, 23% occurred without symptoms 7. The remainder reported chest pain, dyspnea, or had evidence of hemodynamic compromise or arrhythmias. The longest time to discovery of migration was 8 months, although the vast majority (33 of 58 cases) were recognized immediately after stent deployment. Most IVC filter distant embolization cases were described as intracardiac or intrapulmonary, including nine cases of the stent becoming entrapped within the tricuspid valve. In the current literature, there is only one case report of a stent entrapped within the tricuspid valve being successfully retrieved percutaneously. The mechanism of migration is not well established, but some speculate that increasing intrastent clot burden could create a “sail effect,” described as blood flow through the filter inducing cephalad movement, resulting in stent migration 8. The factors that increase the risk of migration include technical problems such as ruptured balloon or underdeployment limiting adequate apposition to the vessel wall.

Descriptions of iliac venous stents, as well as lower extremity venous stents, migrating up to 3 years after placement have rarely been reported 9, 10, 11. Very late stent migration has more often been described in the context of local or systemic trauma, such as self‐massage 10 or multivehicle accident 11. In the case described above, no precipitating event was noted and migration was presumed to be spontaneous.

A few aspects must be considered when determining a management strategy for a migrated venous stent. First and most important is stent location. If the stent has embolized to an area that would be high risk for percutaneous removal (such as embedded within the tricupsid valve), then a surgical team should be involved in deciding a management approach. The severity and acuity of symptoms due to stent migration, patient comorbidities, and available surgical expertise must also be considered. In one published case, a patient with very poor prognosis and high surgical risk was managed conservatively for right heart failure but died 4 weeks after presentation 3. Many factors – from operator experience to patient and stent characteristics – play into the success of percutaneous removal. Owens et al. 12 reported 42 attempted cases of percutaneous retrieval. Of these, 41 of the stents were intracardiac, and only 29 were removed successfully. Conversely, Slonm et al. 13 report 96% success with percutaneous retrieval in 27 cases.

In conclusion, this case illustrates the identification and successful operative management of severe tricuspid regurgitation caused by venous stent migration into the right ventricle. While certain procedural factors (including stent size, stent length, and inadequate stent deployment) increase the risk of this uncommon complication, multiple factors (stent location, patient comorbidities, and stent type) should be considered when determining a management strategy. Although an open operative management has been the traditional approach, percutaneous techniques and device retrieval equipment will continue to improve. In the interim, we suggest that a multidisciplinary team approach that includes general internists/cardiologists, interventionalists, and cardiac surgeons may be warranted when addressing this rare complication.

## Conflict of Interest

None declared.

## Authorship

KH, AB, MF, SH, and MK: contributed to manuscript preparation and editing.
